# European and global approaches to survey ageing populations and perspectives for joint approaches to measure age-related health outcomes

**DOI:** 10.1186/1753-6561-7-S4-I1

**Published:** 2013-08-23

**Authors:** Liv Bode, Christa Scheidt-Nave

**Affiliations:** 1 Department of Epidemiology and Health Monitoring, Robert Koch Institute, Berlin, Germany

## 

Age-related diseases and functional decline put health care systems at a challenge. It is still unclear, whether a compression or extension of morbidity or some sort of balance is likely to be expected. Probably, different scenarios may either coexist or change depending upon both the type of health outcome (particular diseases, multi-morbidity and disability) and contextual factors (socioeconomic, cultural and political). Population-based studies at the national or regional level will provide valid and comprehensive information on age-related changes in population health, as continuous health monitoring will permit analyses of time trends. Prospective studies on lifetime health trajectories are necessary to identify determinants of healthy ageing and critical phases of decline. A premise to achieve all this including cross-national comparability is to establish a consensus platform for harmonising methods, key concepts and indicators.

Against this background, the 2nd European Workshop on Health and Disability Surveillance in Ageing Populations (EUWAP) held at the Robert Koch Institute (RKI) in Berlin, Germany on November 22-23, 2012, pursued two goals, (1) to take stock of existing European and global approaches for surveying ageing populations, and (2) to identify perspectives for joint future collaborations related to cross-national comparisons and the harmonisation of methods and indicators. The presentations and plenary discussions unfolded the large differences which currently still exist regarding concepts, indicators and instruments to assess healthy ageing as well as age-specific aspects of health, e.g. multi-morbidity, frailty, and disability to perform activities of daily living.

In her keynote lecture, Dorly Deeg highlighted the need for a priori harmonising concepts and instruments by illustrating difficulties arising from early post-harmonisation approaches in two European projects, the EU-funded Comparison of Longitudinal European Studies on Ageing (CLESA, 2001-2004)) and more recently, the European Project on Osteoarthritis (EPOSA, 2009-2013). Large EU-funded research consortia such as the Survey of Health, Ageing and Retirement in Europe (SHARE) and the Consortium on Health and Ageing: Networks of Cohorts in Europe and the United States (CHANCES) have taken efforts to harmonise data collection. As reviewed by Simone Croezen and co-authors, SHARE results have been compared with aggregate results from large national or cross-national health surveys, such as nationwide Health Interview Surveys (HIS), the European Health Interview Survey (EHIS), the European Social Survey (ESS), the European Union Labour Force Survey (EU-LFS) and the European Union Statistics on Income and Living Conditions (EU-SILC). Comparisons of SHARE results will be extended to both, aggregate and individual-level data from other national health surveys. CHANCES has implemented a specific work package (CHANCES Health Module), in order to establish a core set of health indicators that provide comparable data between all participating cohort studies. In their presentation, Simone Croezen and Martin Bobak outlined the main concepts and methods applied. The EU-funded research consortium of Collaborative Research on Ageing in Europe (COURAGE) implemented the WHO standard to assess disability and health (ICF; International Classification of Functioning, Disability, and Health). Matilde Leonardi summarised the COURAGE protocol which addresses research, policy and civil society for defining future directions in view of an ageing Europe. A global perspective was provided by Somnath Chatterji who in his presentation illustrated the WHO Study on global Ageing and Adult Health (SAGE). This study has been ongoing for more than a decade using instruments adapted from those of the World Health Survey (WHS) and sixteen other surveys on ageing. SAGE covers multiple domains of health and populations aged 50+ years in six middle and low income countries (China, Ghana, India, Mexico, Russian Federation, and South Africa). Cross-study comparisons with other large studies like SHARE are part of the SAGE concept.

Apart from differences between countries and cohorts, comparisons of results between different studies are hampered by the complexity of health concepts (e.g. multi-morbidity, functional capacity, and disability) and health determinants (e.g. health-related behaviour, quality of healthcare, and socioeconomic context conditions). More importantly, specific components of these concepts are changing over time requiring adaption of indicators and instruments, as Marti Parker emphasised.

Which social, psychological and biological factors are setting the course for healthy ageing already in young age and across the life-time are major questions focused by an inter-disciplinary project on Healthy Ageing across the Life Course (HALCyon) led by Diana Kuh. HALCyon encompasses data from nine birth cohorts in Great Britain. In the context of a life course perspective, health inequalities are one out of seven main priorities worked by Carol Jagger within FUTURAGE, an EU-funded project of 12 EU-countries and Israel which developed a road map for the next decade of ageing research in Europe.

Frailty and disability are important dimensions of health in older people, but are unevenly defined and measured. The above mentioned WHO classification of disabilities (ICF) is mainly focusing on physical functioning. KORA-Age, a longitudinal multi-disciplinary cohort study (region Augsburg, Germany) integrates functioning based on ICF into a comprehensive view on prevalence and determinants of multi-morbidity, disability and successful ageing, as Eva Grill explained. According to the widely used Fried frailty index, three out of five characteristics (weakness, slowness, exhaustion, weight loss, physical inactivity) characterise a person as frail. However, a generally accepted definition is still pending. A modification of the Fried Frailty Criteria introduced by Hermann Brenner initiated a lively discussion. His modified index predicted a higher mortality even among 50-64 year olds based on ESTHER, a cohort study of older adults in Saarland, Germany.

An important indicator of both disability and frailty is slower gait speed. New data from the English Longitudinal Study on Ageing (ELSA), presented by Panayotes Demakakos revealed a bidirectional association between gait speed and depressive symptoms. How changes in self-rated health, health behaviour, social participation and related socioeconomic conditions are interacting, has been investigated in the German Ageing Survey (DEAS). As pointed out by Susanne Wurm, DEAS as an interview-based survey system including repeated nationally representative surveys of persons aged 40+ years, permits comparisons between different birth cohorts at the same age and provides the option to adapt to new indicators every six years. The continuous national health monitoring system at the RKI also permits time trend analyses, although comparisons are still limited to only a few points in time. As exemplified by Christa Scheidt-Nave in her presentation, several health indicators relevant to the population 65-79 years can be compared between the first wave of the German Health Interview and Examination Survey for Adults (DEGS1) conducted in 2008-2011 and the German National Health Interview and Examination Survey conducted more than a decade earlier in 1998.

EUWAP came at the right point in time and with the clear message to strengthen harmonisation efforts. For the future, even closer collaboration and net-working between the different research consortia are intended, in order to facilitate pooled analyses, cross-country comparisons, and validation studies.

Please see Additional file 1 for the programme of the EUWAP workshop.

## Supplementary Material

Additional file 1 EUWAP workshop programme Click here for file

