# Supplementary material for: European and global approaches to survey ageing populations and perspectives for joint approaches to measure age-related health outcomes
Source: BMC Proc. 2013 Aug 23;7(Suppl 4):I1. doi: 10.1186/1753-6561-7-S4-I1 (PMC3892732; doi:10.1186/1753-6561-7-S4-I1)
Supplement: Additional file 1 — EUWAP workshop programme [file 1753-6561-7-S4-I1-S1.PDF]

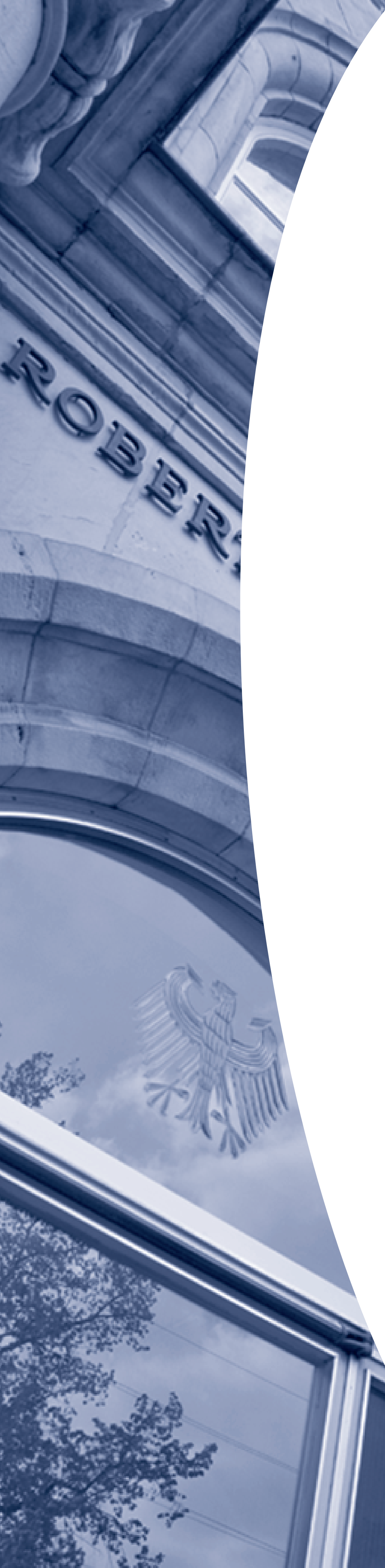

ROBERT KOCH INSTITUT

EUWAP

European Workshop on Health and Disability Surveillance in Ageing Populations

Berlin, November 22–23, 2012

Robert Koch Institute
Nordufer 20
13353 Berlin, Germany

World Health Organization

EUWAP

November 22–23, 2012

Expert Workshop
(EU and WHO)

Harmonizing Concepts, Instruments and Indicators to Assess Healthy Ageing, Multi-Morbidity and Disability in Older Populations

Perspectives for Future Collaboration

Robert Koch Institute (RKI)
Berlin, Germany

Thursday, November 22, 2012

Where we are - European and Global Approaches to Survey Ageing Populations

Programme

12:45–13:30 **Arrival at Meeting Site**

13:30–13:40 **Welcome Address**

Bärbel-Maria Kurth, Robert Koch Institute (RKI), Head of Department of Epidemiology and Health Monitoring, Berlin, Germany

13:40–13:50 **Opening Address**

Christa Scheidt-Nave, RKI, Division of Non-Communicable Disease Epidemiology, Berlin, Germany

13:50–14:55 **KEYNOTE ADRESSES**

13:50–14:30 **Where we are now – Monitoring Health and Disability in Older European Populations and what Harmonization Efforts (CLESA, EPOSA) could tell (keynote lecture)**

Dorly J. H. Deeg, VU University of Amsterdam, EMGO Institute of Health and Care Research, Amsterdam, the Netherlands

14:30–14:55 **WHO-SAGE – Global Ageing and Adult Health with Focus on Low- and Middle- Income Countries**

Somnath Chatterji, World Health Organisation, Multi-Country Studies Unit, Geneva, Switzerland

14:55–17:05 **SESSION I**

European Research Collaboration Programmes

Chair: Judith Fuchs, Juergen Thelen (RKI, Division of Non-Communicable Disease Epidemiology, and Division of Health Monitoring: Surveys and European Collaboration, Berlin, Germany)

14:55–15:20 **SHARE – Survey of Health, Ageing and Retirement in Europe. Validating Health Indicators and Outcomes across Europe**

Simone Croezen, Erasmus Medical Centre, Department of Public Health, Rotterdam, the Netherlands

15:20–15:50	## Coffee/Tea
15:50–16:15	**COURAGE – Collaborative Research on Ageing in Europe. Determinants of Health and Health-related Outcomes in three European Countries**<br><br>**Matilde Leonardi**, Foundation of the Carlo Besta Neurological Institute, IRCCS, Neurology, Public Health, Disability Unit, Milan, Italy
16:15–16:40	**The HALCyon programme – a Life Course Approach to Healthy Ageing**<br><br>**Diana Kuh**, Medical Research Council (MRC) Unit for Lifelong Health and Ageing, MRC National Survey for Health and Development, London, UK
16:40–17:05	**Major Drivers of Health Inequalities in the Elderly and Future Research Needs identified by FUTURAGE – a Roadmap for Ageing Research in Europe**<br><br>**Carol Jagger**, Newcastle University, Institute for Ageing and Health, Newcastle upon Tyne, UK
17:05–17:55	**Plenary Discussion**<br><br>European and Global Perspectives for Harmonizing Concepts and Instruments of Data Collection in Older Populations
17:55–18:00	**Closure of Meeting, Day 1; Organisational Notes**<br><br>**Liv Bode**, RKI, Division of Non-Communicable Disease Epidemiology, Berlin, Germany
20:00	## Dinner Buffet and Get-Together

Friday, November 23, 2012

Where we have to go – Perspectives for Joint Approaches to Measure Age-Related Health Outcomes

09:00–09:10 **Introduction, Day 2**

Christa Scheidt-Nave, Liv Bode; RKI, Division of Non-Communicable Disease Epidemiology, Berlin, Germany

09:10–10:25 **SESSION II**

Input on Indicators from European Studies

Chair: Liv Bode, Markus Busch (RKI, Division of Non-Communicable Disease Epidemiology, Berlin, Germany)

09:10–09:35 **Health Trends in the Elderly – the Need for Multiple Health Indicators to Measure Different Components of Health**

Marti G. Parker, Karolinska Institute, Ageing Research Center (ARC), Stockholm, Sweden

09:35–10:00 **Health Module to Measure Health-Related Outcomes in the Elderly – a Project of the Consortium on Health and Ageing: Network of Cohorts in Europe and the United States (CHANCES)**

Simone Croezen, Martin Bobak; Erasmus Medical Centre, Department of Public Health, Rotterdam, the Netherlands, and University College London (UCL), Department of Epidemiology and Public Health, London, UK

10:00–10:25 **Depressive Symptoms and Physical Functioning in Older Adults: New Findings from the English Longitudinal Study on Ageing (ELSA)**

Panayotes Demakakos, University College London (UCL), Department of Epidemiology and Public Health, London, UK

10:25–10:55 **Coffee/Tea**

10:55–12:10 **SESSION III**

Input on Indicators from German Studies

Chair: Hendrik van den Bussche (University Medical Centre Hamburg-Eppendorf [UKE], Department of Primary Medical Care, Hamburg, Germany)

10:55–11:20 **Epidemiological Approaches to Frailty – Contributions from the ESTHER Research Network**

Hermann Brenner, German Cancer Research Centre (DKFZ), Department of Clinical Epidemiology and Ageing Research, Heidelberg, Germany

11:20–11:45 **Predictors of Successful Ageing, Frailty and Disability based on KORA-AGE**

Annette Peters, German Research Centre for Environmental Health (Helmholtz Zentrum München), Institute of Epidemiology II (EPI II), Munich, Germany

11:45–12:10 **Health in Older Age. Trends from the German Ageing Survey (DEAS)**

Susanne Wurm, Clemens Tesch-Römer; German Centre of Gerontology (DZA), Berlin, Germany

12:10–13:10 **Lunch Break**

13:10–13:35 **Addressing Health and Ageing in the German National Health Monitoring System**

Christa Scheidt-Nave, RKI, Division of Non-Communicable Disease Epidemiology, Berlin, Germany

13:35–14:35 **Plenary Discussion**

Perspectives for Future Collaboration and Joint Research Proposals (HORIZON 2020)

14:35–14:45 **Closing Remarks**

Christa Scheidt-Nave, Liv Bode; RKI, Division of Non-Communicable Disease Epidemiology, Berlin, Germany

14:45–15:15 **Optional Guided Visit of the Robert Koch Museum vis-à-vis the Main Lecture Hall**

Notes

Venue

Robert Koch Institute
Main Lecture Hall
Nordufer 20
13353 Berlin, Germany

Transport connection (www.bvg.de) to »S+U Westhafen«

Arrival by flight

- Airport Berlin-Tegel by shuttle bus (TXL, direction S+U Alexanderplatz via Hauptbahnhof) until stop »U-Turmstr.«, from here by subway (U9, direction Osloerstr.) until station »S+U Westhafen«, from here a few minute walk (25 min in total)

- Airport Berlin-Schönefeld by city train (S9, direction S+U Pankow) until station »S+U Ostkreuz«, from here line S42 until station »S+U Westhafen«, from here as above (70 min in total)

Arrival by train

- Main railway station »Hauptbahnhof« by shuttle bus (TXL, direction Flughafen Tegel Airport) until stop »U-Turmstr.«, from here as above (17 min in total)

Contact
Liv Bode, PhD
Organizer and Scientific Coordinator of EUWAP
Email: bodel@rki.de

Publisher: Robert Koch Institute
Department of Epidemiology and Health Reporting
Division of Non-Communicable Disease Epidemiology
General-Pape-Str. 64–66
12101 Berlin, Germany
Editing: Liv Bode
Layout: Hans-Günter Bredow
Print: RKI Print Office
Issue: November, 2012
